# Low enrolment in Ugandan Community Health Insurance Schemes: underlying causes and policy implications

**DOI:** 10.1186/1472-6963-7-105

**Published:** 2007-07-09

**Authors:** Robert Basaza, Bart Criel, Patrick Van der Stuyft

**Affiliations:** 1 Ministry of Health Uganda, PO Box 27450, Kampala Uganda; 2 Department of Public Health, Institute of Tropical Medicine Nationalestraat 155, B-2000 Antwerp, Belgium

## Abstract

**Background:**

Despite the promotion of Community Health Insurance (CHI) in Uganda in the second half of the 90's, mainly under the impetus of external aid organisations, overall membership has remained low. Today, some 30,000 persons are enrolled in about a dozen different schemes located in Central and Southern Uganda. Moreover, most of these schemes were created some 10 years ago but since then, only one or two new schemes have been launched. The dynamic of CHI has apparently come to a halt.

**Methods:**

A case study evaluation was carried out on two selected CHI schemes: the Ishaka and the Save for Health Uganda (SHU) schemes. The objective of this evaluation was to explore the reasons for the limited success of CHI. The evaluation involved review of the schemes' records, key informant interviews and exit polls with both insured and non-insured patients.

**Results:**

Our research points to a series of not mutually exclusive explanations for this under-achievement at both the demand and the supply side of health care delivery. On the demand side, the following elements have been identified: lack of basic information on the scheme's design and operation, limited understanding of the principles underlying CHI, limited community involvement and lack of trust in the management of the schemes, and, last but not least, problems in people's ability to pay the insurance premiums. On the supply-side, we have identified the following explanations: limited interest and knowledge of health care providers and managers of CHI, and the absence of a coherent policy framework for the development of CHI.

**Conclusion:**

The policy implications of this study refer to the need for the government to provide the necessary legislative, technical and regulative support to CHI development. The main policy challenge however is the need to reconcile the government of Uganda's interest in promoting CHI with the current policy of abolition of user fees in public facilities.

## Background

Community Health Insurance (CHI) is a general term for voluntary health insurance schemes organized at community level, that are alternatively known as mutual health organizations (or *mutuelles de santé *in French) or micro-insurance schemes. They all share the following characteristics: being run on a not for profit basis, targeting informal sector and applying the basic principles of risk-sharing and members' participation in management [1-3]. In Uganda, families can join schemes only as groups. A group is a set of people who are registered in the same community, organization or work place (burial society, cooperative, school, etc) or who live in the same village [4,5]. Despite promotion of CHI schemes in Uganda since the mid 90's, membership has remained persistently low, with only 30,000 or so people enrolled in the schemes, comprising approximately 2% of the catchment population. Moreover, the total number of schemes has not exceeded thirteen. In addition, CHI schemes have only generated very little additional funding: on average, CHI contributions constitute 2% of the overall recurrent budgets in those hospitals where the schemes have been implemented. A study on declining subscriptions in Maliando CHI scheme in Guinea Conakry in West Africa [6] identified poor quality of care offered to members and problems of subscription affordability amongst poor and/or large families as the key reasons for low enrolment. Little is known on the reasons for low enrolment in Uganda, therefore an evaluation was carried out on two selected CHI schemes; Ishaka and Save for Health Uganda (SHU) with the objective of gaining better understanding of this low enrolment.

The article is structured in four parts. First, we introduce the general context of the health system and the situation of CHI in Uganda. Secondly, we present the research methodology. The selections of the study sites, data collection and analysis including design of the guide for case study evaluation are elaborated. In the third step, we present the results. We conclude the paper in the fourth part with a short discussion and point out areas for further research.

### The context

#### Health care provision and policy environment

In Uganda, the health system is made up of both public and private health care providers together with traditional healers. The public system provides sixty percent of all health care services. The private-not-for profit (church related) sub-sector provides about 30% and the rest (10%) is supplied by the private-for-profit sub-sector. Private-not-for-profit units often exist in remote isolated places acting as the sole providers of health care. User fees were introduced into the public health system in the 90's. Amid exclusion of over 50% of the population, in 2001 user fees were abolished in the general wings of public hospitals and health centers but continued to be levied in the private wings. User fees had been introduced to meet the huge public sector deficit and as a response to pressure from structural adjustment programs. However, the private sector has always charged user fees and continues to do so. Significantly, in the context of user fees abolition, out of pocket payments still comprise approximately 54% of overall health care expenditure[7]. The health system is decentralized at district and health sub-district level with no regional tier. However, the policy framework is set by the central government.

#### Development of CHI in Uganda

The first scheme was set up in 1996 at a rural hospital in Kisiizi. The majority of schemes in Uganda are hospital-based, run (and largely owned) by the hospitals themselves, with exception of the Save For Health scheme, which is run and owned by local communities. The schemes were started jointly by the Ministry of Health and various donors. The hospitals carry out primary health care activities and serve as secondary referral centers. External support from donors and the Ministry of Health provided to the schemes comprised of introductory training and assistance in scheme design. Most importantly, in a majority of the schemes, funds were provided for deficit funding and for meeting operational costs like computers, sensitization and purchase of stationery.

#### Local context of the two schemes studied

The first scheme, Save for Health Uganda (SHU) is situated 100 Km north of Kampala and is owned by local communities in Luwero, Nakasongola and Nakaseke districts. Save For Health Uganda (SHU) is a Non-Governmental Organisation (NGO) which operates through multiple independent sub-schemes. The Save For Health scheme was established at the time when communities were recovering from effects of the five-year long civil war of the early 80's. The first sub-scheme was set up in 1999 and by December 2005, a total of 2,840 people were enrolled. Some slight variations in benefit packages, premiums, co-payments and other operational details between the individual CHI constituent sub-schemes included in SHU do exist. One of the measures used to limit adverse selection is a waiting period of three months before new members' can access benefits. Another is a requirement for village-based enrolment comprising a minimum of 100 people. A village has an average population of 1000 people. Kiwoko hospital is the main provider for SHU and is owned by the Anglican Diocese of Luwero. The premium per individual member of SHU amounts to about US $ 2.0 per annum. The schemes have a requirement for a co-payment which varies per sub-scheme. The fee structure at Kiwoko hospital is a mixture of flat fees and fees per service item. The average fee for non scheme members is US$ 3 per outpatient attendance and US$ 15 for an admission. The fees include the consultation, diagnostic tests and drugs. The hospital provides 12% discount of the hospital bill to scheme members. There are 10 sub-schemes, seven of which provide credit while the rest are mixed ie a combination of credit and insurance. Credit schemes offer an arrangement where members contribute money in advance and if one is sick then she/he can borrow from the fund. At the request of sub-scheme members, credit schemes were set up first due to the level of dissatisfaction expressed about the promotion of insurance-based schemes. Later, mixed schemes were established because some groups felt that their members would be unable to sustain credit payments, especially in the case of long illnesses. In mixed schemes, a portion of contributions are placed in an 'insurance-basket' and a portion in the 'credit basket'[8]. This study focuses on mixed schemes with particular emphasis on the insurance element.

The second scheme that was studied is the Ishaka CHI scheme which is owned and controlled by the Ishaka Adventist Hospital and is situated about 350 Km west of Kampala. It was also set up in 1999. The premium is US$ 2 per family member every three months, and a small co-payment of US$0.5 for out patient consultations and US$ 2.5 for every inpatient admission. The benefit package includes all services provided in both outpatient and inpatient departments at Ishaka hospital including drugs and diagnostic tests. Dental and optical cares are excluded. Like SHU, the scheme also operates measures against adverse selection, including a waiting period of two weeks. Another measure is a group-based enrolment requirement; 60% of the group must enroll before the scheme becomes operational. User fees are a mixture of flat fees and fee-per-service item. Non scheme members pay an average of US$5.00 for a consultation, drugs and diagnostics for an out-patient case and similarly US$15.00 for an inpatient case.

Both Ishaka and Kiwoko are church-owned facilities and the only not-for-profit general hospitals in their catchment area. There exists public health centres that do not charge and private for-profit health centres in the hospitals' catchment areas. Referral to higher level care hospitals does not form part of the benefit package in either scheme. Immunization and family planning are provided free-of-charge to both scheme and non-scheme members. Both schemes are also involved in health promotion, for example the use of insecticide treated mosquito nets which are sold at subsidized prices to scheme members. Anti-retroviral drugs for HIV/AIDS are provided free of charge by these two hospitals to both scheme and non scheme members. Scheme members with opportunistic infections receive treatment as part of the CHI benefit package. The features and trends in enrolment of both schemes are presented in table 1. The principle source of revenue for the majority of the communities in both schemes is subsistence farming. Both banana growing and livestock keeping are commonly found in the catchment area of Ishaka. Besides livestock keeping amongst members of the SHU scheme, local communities grow coffee beans, but this has been devastated by coffee wilt disease. This blow greatly affected the communities' incomes.

**Table 1:** Features and trends of the Ishaka and SHU schemes

	*Ishaka*	*SHU*
Type	Provider-driven	Community-run
Premium per annum per person (US$)	8	2
Co-payment OPD (US$)	0.5	Varies per sub-scheme
IPD (US$)	2.5	
Benefit package	Inpatient and outpatient care at Ishaka hospital	In-patient and out patient care at Kiwoko hospital
Exclusions	Chronic diseases, dental and optic care	Chronic conditions
Yearly coverage*		
2002	1163 (2%)	824 (<1%)
2003	1339 (3%)	1593 (3%)
2004	1106 (2%)	2156 (4%)
2005	970 (2%)	2840 (6%)

* The percentages in the table reflect the CHI coverage of the population living in the hospital catchment area.

## Methods

The research was carried out in the period November 2004–December 2005. A case study research design was chosen in order to permit an in-depth focus on relationships and processes that may help explain the low levels of enrolment in CHI schemes. Specifically, the research intended to explore factors on both demand and supply side of health care delivery that can explain this low enrolment. A variety of sources, data types and research methodologies were employed, including a review of records, key informant and exit interviews. Triangulation provided an opportunity to corroborate findings and to enhance the validity of the data. The research team reviewed the scheme feasibility studies, annual reports, membership data, annual reports of the umbrella association – the Uganda Community Based Health Financing Association (UCBHFA)- and policy documents from Ministry of Health headquarters.

### Design of a guide for the case study evaluation

Based on existing frameworks for the analysis of CHI schemes in sub-Saharan Africa, we developed a guide for our case study evaluation. In a first instance we carried out a cross cutting analysis of existing frameworks for the study of CHI (table 2). The frameworks were selected on the basis of their comprehensiveness and on their systems approach to CHI [9-13]. The analysis focused on six different dimensions: (i) the CHI typology used; (ii) the identity of the main actors involved in setting up the scheme; (iii) the nature of the relationship between the key players; (iv) the flow of funds from payers to providers; (v) the benefits package; and (vi) the role of the public health authorities in CHI schemes. Eventually a synthetic framework for the case study evaluation of Ugandan CHI schemes was established based on review of these existing frameworks (table 3).

**Table 2:** Framework for analysis of CHI schemes in sub-Saharan Africa

**Name of the framework**	**Features**					
	*Typology used*	*Main players*	*Relationship between key players*	*Flow of funds*	*Supply and utilization of care*	*The role of Public Health Authority (PHA)*

Arhin-Tenkorang D 2001	Mutual benefit society, provider insurance, mutual partnership and third party insurance	External stakeholders (members, suppliers etc); internal stakeholders (employees, managers etc).	There is exchange of resources and expectations between stakeholders within the scheme.	From subscribing unit to agency/insurance scheme and then provider	Presence of manuals, guidelines for quality assurance and contracts between the insurer and provider or client and insurer	Supervision of a range of benefits, financing of benefits, instituting and enforcing regulations. Linkages of CHI schemes with the formal financing networks.

Hohmann J et al 2001	Not described	External stakeholders, target groups (individual, household or group), insurer or other sub-contracted providers	Target group make regular contributions to an insurance scheme. A benefit package is provided by the scheme directly or by contracting other sub-contracted providers. Instead of or in addition to, the scheme, may reimburse claims	Funds flow from the target group to the insurance scheme and to the provider.	The benefit package is defined by regulations or by requirements of subscribers. The schemes may provide other products like cash benefits, burial and harvest insurance	Supervising and regulating of the schemes. Registration/licensing of the insurance schemes. Accreditation guidelines. The PHA may be in favour of the schemes or against, whilst different ministries may have different opinion.

Criel B. 2000	Mutual – provider driven.	Subscribing unit, insurer or purchaser and the health care provider.	Two explicit relationships: (1)Between the subscribers and insurer. The insurer is an intermediary between the subscriber and the provider or the subscriber may deal with the insurer who may at the same time be the provider (2)Insurer and provider: may have a contract or a convention	Three main categories: (1)Contributions which are mainly premiums to the purchaser (2) payment of the provider by the insurer (3) payment to the provider at the time or point of use	The content of the package is crucial. There may be other benefits outside the health system such as transport	Technical & regulative control, legislative and funding role. Social animation role in line with PHC philosophy

Musau S 1999	2 types of schemes identified: A) Covering high costs, low incidence health care events B) Those with low cost but probability events	Members, providers and insurers.	Highlights the advantage of enrolling cohesive communities as vehicles for development of CHI.	Members pay a premium to providers or mutual organizations which pays the providers	Benefit package defined in the guidelines.	Policy and legal frame work, a regulatory one and finally technical support

Bennett S, Creese A and Monasch R1998	Two typologies presented. One based on health facility, community, co-operatives or mutual, NGO and Government The second one based cost: Type I: high cost and low frequency events (hospital inpatient care) and Type II: low cost and high frequency (basic primary care).	Government, NGO, communities and providers.	Membership can be geographical or place of residence or place of work. Individual enrolment is subject to adverse selection.	In type 1, premiums are paid to the scheme and the scheme pays the hospital on case-basis or fee per service item. Type II premiums are simply allocated to the nearest provider on a lump sum basis.	Benefit package may be available at the facility or published defined lists and or financial ceilings.	Provide policy framework and operational guidelines, training of community members in scheme management and ensuring accountability of fund holders. Could provide a subsidy as well.

Shaw R and Ainsworth M 1995	- Nation – wide schemes - District based Schemes -Small facility or village based schemes	Members, providers of care and third party.	Subscribing unit could be an individual or a family or a household. In order to minimize adverse selection, group insurance should be promoted.	Funds are collected during the harvest/high income season to the facility or government. The facility provides the care.	Within the pool benefits are provided on the basis of need rather than income. No further elaboration.	Not overseen

**Table 3:** Guide for the case study evaluation of the Ugandan schemes

*Feature*	*Key issues*
**Role of public Health Authorities (National and District levels)**	Policy, strategic framework and regulation, setting guidelines for accreditation of providers/insurers, specific roles of Ministries other than health, technical support in the design of schemes, promotion and marketing, funding role and subsidy.

**Scheme design**	Problems encountered in the set up period, objectives of the scheme, target groups, enrolment period, unit of enrolment, benefit package marketing, monitoring and evaluation, management information system, premiums and co-payment, risk management, cost escalation and any other services offered by the scheme.

**Scheme members**	Awareness of CHI, reasons for joining the scheme, involvement in CHI, subscribing unit and previous experiences with community financing initiatives.

**Non scheme members**	Awareness of CHI and reasons for not joining the scheme

### Selection of study sites and data collection

Two cases out of a total of thirteen existing schemes in Uganda were selected. The Ishaka scheme was chosen because it reflects the typical design of most Ugandan schemes, i.e. a model where the provider is also the insurer. On the other hand, the Save for Health Uganda (SHU) scheme operates completely differently. Save for Health Uganda is a CHI model which is based on member-based organisations that mediate between households and provider. The scheme collects money from householders and then contracts the provider to supply specified services. The scheme membership is representative of almost all the country's ethnic groups.

Both semi-structured key informant and exit interviews were carried out in both schemes. The characteristics of the persons interviewed and duration are presented in table 4. A total of 62 individuals were recruited for Key Informant interviews (KI) at National and district and Exit Interviews (EI) at hospital levels (23 KI interviews and 39 EI). The research team carried out exit interviews with all the scheme members who visited the hospitals during the period of data collection. Selection of non scheme members involved every second exit patient who qualified as a non-scheme member from within the catchment area the hospital. Fieldwork accounted for eight days for each of the two schemes, analysis of the results lasted four days, transcribing four days, thus the total duration of data collection for the two schemes was twenty four days. The interview processes were tape recorded and additional notes taken. Three researchers conducted the interviews. They were social scientists and received four days training which involved familiarisation with the principles of a case study evaluation and other research methodologies employed. In addition, this training included an introduction to the basics of CHI. Pre-testing of the topic guide was done on another scheme, the Kitovu CHI Scheme in Kitovu Hospital, Masaka District. This scheme was chosen because: (i) it represented the typical design of the majority of schemes; (ii) it is sited near Kampala, the base of the study; (iii) it has good record keeping standards and (iv) the hospital officials are receptive to researchers. All the exit interviews were carried out in local languages (*Runyankole* for Ishaka and *Luganda* for SHU scheme) after the patients had received treatment. The key informant interviews were conducted with the umbrella association of Ugandan CHI schemes, the Uganda Community Based Health Financing Association (UCBHFA). In addition, Ministry of Health (MOH) Planning Department officials were interviewed, in particular those responsible for health financing. These were the Commissioner of Health Services responsible for planning plus three Senior Health Planners. Out of the four staff interviewed, two were used exclusively to validate responses. Bilateral donor agencies like the United States Agency for International Development (USAID), the Department For International Development (DfiD) and different religious bureaux who play or played an active part in the inception of schemes were also interviewed. At district level, individuals interviewed were the District Director of Health Services (DDHS) and the Secretary for Health (the elected head of health services) in the two districts where the schemes are located. Permission was also sought for their involvement in the research and for the use of tape recorders as well.

**Table 4:** Characteristics of persons interviewed

**Key informant interviews**	**Numbers**
Hospital and scheme level (Medical Directors, Superintendents, other managerial, scheme staff).	9
District level (DDHS, Secretaries of Health).	3
National level Health Planners, Development Partners, WHO country office, Religious Bureaus, UCBHFA staff, average duration in post 5 years	11
Duration of each interview 30 minutes	

**Exit interviews from both schemes**	

Patients who are scheme members Average duration as scheme members 3–5 years, duration of each interview 45 minutes	28
Patients who are non-scheme members, duration of each interview 15 Minutes, (6 from Ishaka and 6 from SHU)	12

**Total**	**63**

Transcription and translation of the EI to English was carried out on the same day by the research team. Also transcription of KI interviews was done on the same day of the interviews. The framework method was used for the data analysis [14]. Indexing and analysis were completed along five lines: (i) cross analysis between explanations for low enrolment based on reviewed frameworks; (ii) classifications of reasons for joining or not joining; (iii) classification along a range of comparisons made by scheme and non scheme members; (iv) two levels of governance: the central government and the district level; (v) cross comparison of the two schemes, Ishaka and SHU. The findings wherever possible are supported with verbatim quotations from interviewees. Ellipses are used to denote missing speech. For those responses which were quantified, the figures given indicate the number of direct quotes that were collected. The quotes in the text are followed by an index in the bracket and this indicates the key informant or exit interview from where they were collected.

### Ethical approval

Explicit consent was obtained from interviewees. The research team pointed out to the interviewees that the information provided would only be used to develop a national policy on community health insurance. In addition, interviewees were given an option of walking out of the interview session if they so wished. More importantly, this study is part of the work program of the Ugandan health sector approved by government, donors and all stakeholders and put in the second Ugandan health sector strategic plan.

## Results

The data uncovered key issues of policy ranging from the establishment of new schemes, scheme management, reasons for joining/not joining schemes to familiarity and understanding of the scheme amongst its members and promoters. It also highlighted key differences between the two types of schemes. The key results of the interviews are presented in table 5.

**Table 5:** Reasons for low enrolment*

	**Key informants**		**Exit Interviews**		**Total**
		
	*National and district level*	*Scheme and Hospitals*	*Patients (Ishaka)*	*Patients (SHU)*	
Members not deciding on the benefit package	1	-	14	-	15
Lack of information and poor understanding of CHI scheme	1	2	5	2	10
Incapability to raise the contributions (premium)	-	2	4	3	9
Lack of accountability by the scheme managers	5	-	-	-	5
Requirement of teaming up with other people	-	-	2	2	4
Lack of trust in local financial systems		1	1	2	4
Lack of a policy frame-work for CHI	4	-	-	-	4
Abolition of user-fees	2	-	-	-	2
Communities used to free things		2	-	-	2

*Only when directly mentioned as reason for low enrolment

### Policy concerns

At the central level of the Ministry of Health, the KI interviews indicated absence of a central, strategic policy on CHI. Despite this oversight, CHI is explicitly mentioned in health policy documents and the health sector strategic plan including the health financing strategy as one of the financing mechanisms.

"CHI is mentioned in the Health Financing Strategy and the Sector Strategic Plan (KI)".

"No policy yet but CHI is a component of the Ministerial Policy Statement (KI)".

"The Ministry does not have a CHI policy or guidelines (KI)".

At the time of setting up the schemes, there was limited expertise on CHI within the Ministry of Health and amongst donors. There was little or no practical experience in setting up CHI schemes. Currently there exists no regulatory framework for CHI schemes in Uganda. Ugandan schemes do not have specific procedures for the accreditation of providers and insurers for CHI schemes. Schemes do not have safe guards against 'skimming'. The other institution involved in the administration of CHI is the Ministry of Internal Affairs. This Ministry maintains a register of non-governmental organizations involved in CHI. The majority of the schemes begun in the 90's with both financial and technical support from the UK bilateral aid agency to Uganda i.e. the Department For International Development (DfiD) to UCBHFA and, through the medium of the association, indirectly to the schemes. Support from the DfiD ended in 2002. Today the schemes receive no direct financing from governments or donors. No elaborate promotion or marketing plans for CHI exist. Both schemes do not have health care subsidies for the poorest sectors of the population. The government, donors and UCBHFA were and still are involved in basic management training and programme to raise community awareness. This is being done through seminars, sensitization workshops, radio programs and the promotion of mosquito nets.

"UCBHFA does organize some training and experience sharing workshops (KI)".

One notable policy conflict is the promotion of CHI on one hand and the 2001 abolition of user fees in public units on the other hand. Abolition of user fees has provided little impetus for health workers, donors and other stakeholders to promote CHI. The abolition of user fees affected marketing of CHI schemes and obviously jeopardized the policy relevance of CHI in the public sector.

"Hardly any marketing of CHI is carried out because of the abolition of user fees (KI)".

"No policy nor any guidelines on promotion of CHI amid absence of user fees in government units (KI)".

### The setting up of CHI schemes

Feasibility studies were carried out in both Ishaka and SHU schemes prior to launching of the schemes. The explicit objectives of setting up the Ishaka scheme were first to devise a mechanism to reduce hospital debts and second to increase community access to health services.

"There were very many hospital debts and patients could not afford admission (KI)".

"It is very hard to provide services to poor people (KI)".

"The hospital wanted to reduce debts and raise income for running services (KI)".

Different objectives applied to the SHU scheme, which is a community-owned and where the promoters wished primarily to increase access to the services of the local private not-for-profit hospital.

"It was out of notice that patients could not afford hospital bills to the extent of selling property (KI)".

Whereas donor support was instrumental in the setting up both schemes, the Ugandan government played no role in setting up SHU. The Ministry of health with DfiD provided technical support to the Ishaka scheme. In this context it is worth mentioning the unwillingness of some communities to pay for social services. There was a deliberate effort to rehabilitate social services in Luwero during the 90's. This followed an influx of non-governmental organizations which were established to provide free health care services as a result the previous civil war. Many communities became used to provision of free care by various organizations and, as a result, many individuals were no longer willing to prepay for health care.

.... "The communities were used to free things and it took time to appreciate prepayment of health services (two KI)".

This was not a feature seen in the Ishaka scheme.

The public health authority does not monitor scheme in terms of performance or patient follow up in both schemes.

"Nothing is done to ensure that fund managers account to scheme members (KI)".

"The schemes are not regulated by any organization (KI)".

### Community role in the running of the schemes

Two distinct but consistent responses were a feature of both schemes in respect to the role of the community. In the Ishaka scheme, the hospital takes responsibility for all major decisions concerning the scheme. A majority of the respondents in the Ishaka scheme took the view that the decision to determine the basic package, the premium and the co-payments were made by the hospital and scheme management alone.

Out of 17 Ishaka scheme members interviewed, 14 pointed out that they do not have any role in the management of the scheme and made it clear that, in their view, it is the hospital which takes all management decisions.

"The scheme is under the control of the hospital and the communities have hardly any say in running of the scheme (KI)".

The rest of the interviewees (3) were unaware of their role in the management of the scheme.

"It is only our group leader who knows what happens in the scheme (two EI)".

"Almost all the people in our village were registered by relatives and are not aware of the role they are supposed play in the scheme (EI)".

This is different in SHU where the decisions are taken jointly by the scheme members and by the hospital concerned. All the respondents were aware that it is communities who decide on which services will be provided under the scheme and the corresponding level of contribution in the form of premiums. Interviewees got to know the scheme through sensitization by the scheme staff, scheme members and local churches. A majority of the SHU scheme members interviewed were involved in the mobilization of scheme members and decisions on the package of benefits.

"It is we who decide on the type of services to pay for and it depends on how much we are able to contribute as scheme members (7 out of 9 direct responses)".

### Reasons for joining the scheme

The major reasons for joining both CHI schemes were to make it easy to access health care, receive subsidized and prompt treatment.

"I do get affordable good health care paid for in a convenient way ....we are handled nicely....the lines are short...... we get good medicines (EI)".

"When I fall sick I know that I am already covered (EI)".

"To get cheap and affordable mosquito nets (EI)".

Besides these benefits, some respondents in the Ishaka scheme revealed that other reasons for joining the scheme were:

"To get free lunch and transport during meetings! (EI)".

"The scheme has helped us to get to know each other (two, EI)".

Premiums are paid in cash and members said that payment by installment was an important enabling factor. Some scheme members thought that they sought care more frequently than non members

.... "because we are able to pay the co-payment (EI)".

....."because I can afford it through the scheme (two EI)".

However others thought they seek less care:

"I have to visit the hospital less frequently because I receive adequate treatment when ill... (EI)".

"I have to visit the hospital less frequently because previously we received mosquito nets at a low cost (EI and EI)".

A majority of respondents in both schemes would recommend other people to join the scheme in order that they can also access health care conveniently.

Some new members reciprocated this favor and invited others to subscribe in the same way that they were initially invited themselves (16 out of 28 direct responses).

"To be insured and live without worry (EI)".

"Yes, because it has helped me very much (EI)".

"Yes, for them to benefit and be helped in case of illness (EI)".

A minority felt that they would not invite other people to join:

"I would not recommend other people to join because members who fall sick tend to forget to pay their dues when they recover and this impinges on scheme funds (EI)".

"It is the responsibility of the scheme leaders to invite other people to join the scheme, not me (EI)".

Most of non-scheme members confirmed having been provided with information about the schemes.

A majority (14 out of 16 direct responses) of non-scheme members interviewed was aware of the existence of CHI schemes and had received information and knowledge about the scheme from existing members (12 out of 17 direct responses). Other sources included ward posters and word-of-mouth from scheme staff.

### Reasons for not joining the scheme

Key reasons for not joining the scheme were articulated principally during exit interviews with non-scheme members. All non-scheme members interviewed were aware of the existence of both CHI schemes. Their sources of information about CHI are similar to those of scheme members.

One of the commonest reasons for not joining was an incapability to raise the contributions (8 out of 10 direct responses).

"The care given to us at the hospital is good but we can not afford joining the scheme (EI)".

Some other reasons were the worry about joining as a group.

"It requires teaming up with many homes which isn't easy to achieve (EI)".

"I wasn't bothered since I am young and not likely to fall sick (young adult, EI)".

Non-joiners to both schemes offered much the same explanations. Surprisingly, quality of health care provided was never mentioned as reason for either joining or not joining the schemes. Many communities associated with both schemes failed to receive appropriate information on CHI and in a few instances where relevant information was received, the concept was not understood.

"It is a new concept in health care provision and it took a long time to be appreciated by the communities (KI)".

Lack of adequate information about CHI was frequently cited as one of the reasons for not joining a CHI scheme. It was cited by 7 out of 12 non-scheme members interviewed.

......" I did not have adequate information about health insurance (KI)".

"We were not informed about registration timetable (EI)".

"I had not bothered because I did not see the importance of the scheme (KI)".

"We had only one meeting in my village about the scheme. It was even long time ago (EI)".

"The dates of registration are always rushed (EI)".

### Previous experience with local financial associations

Financial institutions throughout the country were distrusted. Ugandans suffered from the closure of various banks and building societies in the 90 s. There was a countrywide collapse of co-operative societies, non-governmental organizations and local groups involved in the credit unions. Some of the local Non Governmental Organizations (NGO) took money from communities with the promise of subsequent assistance which failed to materialize.

"There was the problem of fake NGO's like COWE and it took time for the schemes to regain the community's confidence.... (KI)".

"I fear joining groups because of previous theft of contributions by the owners of the organizations (EI)".

"We could not immediately trust the scheme, even if it was from the church because of previous experience with our local societies (EI)".

### Comprehension of CHI by communities and health professionals

The answers revealed a poor comprehension of the notion of community health insurance by health workers, administrators and health planners. Some interviewees at central level felt it better to promote good management of the health sector budget rather than to invest in CHI.

"CHI schemes do not provide a comparative advantage and so the government is putting money into health facilities (KI)".

"I am not sure that the schemes do help to increase access to health services instead the government should increase the sector budget (KI)".

" The idea of CHI schemes is still new to us and not enough research has been carried out (KI)"

At district level, the initial findings suggested that the political heads of health services were frequently unaware of the existence of CHI schemes or had only limited experience of working with such schemes. One Director of District Health Services interviewed was knowledgeable about the operations of the schemes whereas another one was simply not aware of their existence and usefulness in his district.

"I am yet to catch up with the role of the scheme in my district, leave alone what is CHI (KI)".

"The hospital staff had limited understanding of the way health insurance works and lacked the capacity to manage insurance schemes (KI)".

"There has not been any information on CHI, not even pamphlets, booklets or guidelines (KI)".

"The idea of schemes is very new and the communities and staff need more time to understand the whole concept (KI)".

One respondent suggested that a complementary role could be played by the Ministry for Agriculture in the form of mobilizing and empowering communities to join a scheme. Currently, public health authorities have no direct involvement in the schemes.

## Discussion and conclusions

Based on the results of our two case studies, we forward a set of non-mutually exclusive reasons to explain the current low levels of enrolment in Ugandan CHI schemes. On the demand side, we see the following elements:

i. Difficulties for existing community groups to raise 60% of the membership or 100 families per village prior to enrolment;

ii. Low level of community involvement in the management of hospital-based CHI schemes;

iii. Lack of information on and poor understanding of the notion of community health insurance;

iv. Lack of trust in local financial organisations after previous depressing experiences with similar institutions;

v. And finally, problems in the ability to pay the premium.

However, there was no evidence presented that poor quality health care had contributed to low levels of enrolment.

Turning to the supply-side, we have identified the following explanatory reasons for the disappointing levels of enrolment:

 The absence of a coherent policy framework to promote CHI amidst a backdrop of user-fee abolition in the public sector;

 A lack of information, poor interest in and understanding of the notion of CHI by health professionals (health workers, district services managers and health planners).

At district and national level, the major concerns were the lack of national policy guidelines on CHI schemes, more than operational issues like mobilization. The respondents from the community run model of CHI (the SHU scheme) were able to make a choice on the package and on the level of contributions for the scheme. They were also involved in the recruitment drive. This is different from the provider (hospital) based model, the Ishaka scheme. The ownership of the latter scheme is by the hospital and scheme members see themselves playing a limited role in mobilization and deciding on the contents of the benefit package. Notably, the interviewees at central level also expressed concern over lack of a frame work for accountability by scheme managers in a hospital driven model. This could be part of the explanation for the higher coverage of the SHU scheme (Table 1).

Our study points to a series of obstacles in the development of CHI that have, to varying degrees, also been identified in other studies carried out elsewhere in Africa. Musau (1999) in his study of CHI schemes in East Africa pointed out that the new character of the CHI concept was a contributing factor to the low enrolment in CHI [15]. In the same study the use of the rule of 60% group enrolment before being allowed to access benefits was referred to as one of the measures against adverse selection in the Kisiizi CHI scheme. However, Musau did not accentuate the limitations this rule imposed on the expansion of enrolment into CHI schemes. In this case study quality of care does not appear to be a major issue. This may be explained by the relatively high quality of care provided by church-based hospitals in the two cases investigated compared to the quality in public hospitals [15]. Another study, also using in-depth interviews supplemented by focus group discussions, conducted in the Nouna Health district, Burkina Faso, West Africa [16] demonstrated that a lack of adequate knowledge and understanding in respect of key features of the scheme, poor quality of care, and general distrust of the proposition went a long way to explain low levels of enrolment. There was one similarly-structured study on the Community Health Fund (CHF) in Hanang district, Tanzania in East Africa [17] which also employed focus group discussions and semi-structured interviews. This study concluded that the explanation for low enrolment were the inability to pay the subscription fee, poor quality of care, poor education and limited mobilization of community members to join. In our study, inability to pay comes out as one of the major reasons for failure to enroll into the scheme. The comparative advantages of CHI to the health system in a resource constrained environment have to be demonstrated in order to convince all the stakeholders.

In the different frameworks we reviewed in this research, the role of the public health authorities in the development of CHI was systematically highlighted. The government is to provide policy, legislative, technical and regulative support and control. Additionally, it should be a financier of CHI schemes especially for the indigent. However, the Uganda authorities have subdued this function and have no clear policy and implementation guidelines. The role of government in capacity building, especially in training, has not been taken on board seriously.

But probably the main challenge to be tackled is the following policy dilemma: how to reconcile the government of Uganda's interest in promoting CHI with a political decision to abolish user fees in public health units? There appears to be a major conflict of interests which needs to be cleared out. This situation is further compounded by the fact that abolition of user fees in government facilities certainly does not mean that out of pocket payments have disappeared altogether. District staff operate in ambiguous policy environment and as such they can not promote CHI on one hand and on the other hand be obliged to advocate free health care. A priority for the Uganda government therefore is to establish a consistent policy framework that will unambiguously situate the relevancy and role of CHI in the national health system. Is there a place for CHI in the Uganda national health system? And if there is, what and where is it? One of the elements in this complex decision-making process is the fact that the overall design of many Ugandan CHI schemes, as well as the possibilities for people to participate in deciding on the scheme's design and management, need to be rationalised and improved – as was shown in this study. The modestly better performance of the SHU scheme, at least as far as coverage and member satisfaction are concerned, indicate that there is scope for improvement in many of the other Ugandan schemes. The effect of such corrective measures, implemented in the different schemes, would first need to be closely monitored and assessed before a final decision can be made on the fundamental policy question whether governments should either support CHI schemes or invest in strengthening existing 'free of charge' public health systems.

## Competing interests

The author(s) declare that they have no competing interests.

## Authors' contributions

RB participated in conception and design of the study, data collection, analysis and interpretation of data, drafting the paper and revising the article. BC participated in conception and design of the study, data collection, analysis and interpretation of data, drafting the paper and revising this article whereas PVDC participated in conception and design of the study, interpretation of data, drafting and revising the paper. All authors read and approved the final manuscript.

## Pre-publication history

The pre-publication history for this paper can be accessed here:


